# An Arduino-Based, Portable Prototype for the Recording and Analysis of EEG Signals to Support Self-Detection and Self-Monitoring of Stress

**DOI:** 10.3390/s26113410

**Published:** 2026-05-28

**Authors:** Stamatios Baltzis, Gerasimos Pagiatakis, Nikolaos Voudoukis, Andreas Papadakis, Leonidas Dritsas, Dimitris Uzunidis

**Affiliations:** 1 Department of Electrical & Electronic Engineering Educators, School of Pedagogical and Technological Education, 15122 Maroussi, Attica, Greece; 20sta.mpal@elec.aspete.gr (S.B.); pagiatakis@aspete.gr (G.P.); apapadakis@aspete.gr (A.P.); dritsas@aspete.gr (L.D.); 2 Department of Electrical & Computer Engineering, National Technical University of Athens, 15772 Athens, Attica, Greece; 3 National Center of Scientific Research “Demokritos”, 15341 Athens, Attica, Greece; duzunidis@iit.demokritos.gr

**Keywords:** electroencephalogram, brain signals, alpha signal, beta signal, stress, Arduino

## Abstract

This article describes a portable Arduino-based prototype for the recording and analysis of electroencephalogram (EEG) signals associated with anxiety situations. The system’s main aim is to enable the user to self-detect stress and take self-regulating/relaxing actions in real time before stress escalates. The recorded EEG signals are first processed in the analog domain (including amplification and noise reduction) and then, by using an Arduino Uno board, they are converted into digital format and transmitted through either a wired or wireless connection to a computer to be depicted in both the time and the frequency domains by means of an open-source software. During the performed tests, the system successfully showed visible changes in the alpha and beta brain signals corresponding to the states of resting, induced stress, and the subsequent self-regulation/relaxation process. The proposed prototype (though non-clinical in its present form) has the merits of relatively low cost, easy self-use (outside clinical environments), and real-time EEG signal depiction, and, apart from enabling the user to self-detect and self-monitor stress, it can also be used for educational and/or research purposes.

## 1. Introduction

Anxiety disorders (such as stress) are a frequently occurring issue that, if not properly treated, may severely affect the quality of life of the involved individual [1,2]. On the other hand, anxiety disorders are associated with particular brain signals, such as the alpha, beta, and theta ones (Table 1), that, if recorded through the electroencephalogram (EEG), can provide an objective means for measuring and evaluating anxiety disorders. In this context, the analysis of EEG signals can help in devising biofeedback mechanisms that may enable the involved person to mitigate and even self-treat such disorders.

An important issue regarding electroencephalography is the requirement for a clinical environment and the relatively high cost of the EEG equipment. Taking the above facts into account, this article presents the design and implementation of a low-cost Arduino-based prototype made up of easily available hardware and open-source software, capable of recording and depicting the alpha and beta brain signals with the aim to timely detect stress. The system is suitable for personal use in non-clinical environments and can help in setting up a biofeedback process, based on self-regulation and relaxation and capable of supporting self-monitoring and mitigation of stress in real time. After the extraction of the brain signals by means of EEG and their subsequent analog processing (including amplification and noise reduction), the system employs an Arduino Uno board for the digitization of the analog EEG signal, which is then depicted in both the time and frequency domain by means of the open-source BrainBay software.

The main reason for employing an Arduino board in the proposed system is that Arduino modules are suitable for the development of low-cost, low-power devices that can interact with their environment and exchange information over the Internet. Built up with an 8-bit or a 32-bit Atmel microcontroller, Arduino modules can be easily programmed and reprogrammed by using variants of the C or C++ language in the Arduino integrated development environment (IDE) [3,4]. Nowadays, Arduino boards are at the heart of numerous systems and applications, ranging from weather and environmental monitoring to laser-cutting machines (e.g., [5,6]). A detailed literature review regarding Arduino boards, prototyping with Arduino, and fields of applications is included in [4].

Of the Arduino variants, the most popular ones are the Arduino Uno, Due, Nano, and Mega. In this work, the Arduino Uno (R4) WiFi board was used due to its simplicity as well as the fact that its technical features (memory, processing power, and input/output ports) were adequate for the implemented system.

Regarding electroencephalography and the extraction and analysis of brain signals, several systems have already been proposed. For example, in Ref. [7], a 16-channel EEG system is described that, after the analog processing of the EEG signal (involving amplification and noise reduction), uses an Arduino Mega board to digitize the EEG signal and further process it through an EEG analyzer. The analysis of EEG signals by means of an ATMega microcontroller and the LabView software (using methods such as the continuous-wavelet and the fast-Fourier transforms, as well as the probability distribution function and the peak plot) is presented in [8]. A low-cost Arduino-Uno-based device for measuring EEG signals is proposed in [9], while Ref. [10] focuses on the removal of artifacts created by the power grid, electrooculography, and electrocardiography (commonly present in EEG signals) by means of adaptive filters in cascade. Finally, Ref. [11] presents a review on cutting-edge EEG signal processing techniques focusing on noise reduction, artifact removal, and feature extraction, and also presents current trends regarding EEG signal analysis, such as graph signal processing, deep learning, and real-time processing.

With regard to the association of EEG signals with human state, Ref. [12] attempts a real-time EEG-based recording of cognitive activity (exploiting deliberate eye blink) for control application via an Arduino board. Reference [13], in turn, presents a wearable EEG sensor prototype employing an Arduino board and the LabView software with the aim to overcome the difficulties caused by the need for the EEG tests to be performed in clinical environments.

Regarding stress analysis, Ref. [14] presents an EEG-based method that employs the alpha, beta, and theta brain signals, more specifically the alpha/beta and theta/beta ratios. Reference [15], on the other hand, uses the information obtained by EEG for the classification of long-term stress, while Ref. [16] attempts a review of EEG-based methods for mental stress assessment. Finally, Ref. [17] proposes a wearable EEG-based brain–computer interface for stress monitoring.

Compared to the systems mentioned above (in particular, the system described in [7]), the proposed prototype is fully battery-powered and has enhanced filtering capabilities (e.g., a second 50 Hz notch filter) and the possibility of wirelessly transmitting the EEG data. Additional merits of the presented system are its portability and easy self-use outside clinical environments that allow the user to detect and self-monitor his/her stress and, by means of biofeedback actions (including self-regulation and self-relaxation processes), to mitigate its effects in real time. The system is fully evaluated with regard to its circuit characteristics, cost, power consumption, and operating time and is also treated as an educational tool associated with the STEAM and TPACK frameworks. It has to be noted that, in its present form, the proposed system is a non-clinical one in the sense that its main objective is to support EEG-related experimentation and interpretation of the derived results for educational purposes rather than serving as a diagnostic or therapeutic device.

In this work, stress-related EEG monitoring is mainly based on changes in the alpha-band power, the beta-band power, and the alpha-to-beta power ratio due to the fact that alpha-signal- and beta-signal-related indicators are commonly used in EEG-based stress and cognitive load studies. In general, reduced alpha activity may be associated with increased cortical activation or reduced relaxation, whereas increased beta activity may be related to alertness, attention, and cognitive effort. However, these indicators should not be interpreted as diagnostic biomarkers since their interpretation depends on the recording condition, electrode location, participant state, and possible artifacts. Given that, the proposed system uses these metrics only as supportive indicators for self-monitoring and educational/research purposes.

Regarding localization, frontal and central regions are often involved in cognitive effort and stress-related processing, while parietal/occipital regions are strongly involved in resting alpha activity, especially during eyes-closed conditions. Therefore, the interpretation of alpha and beta changes should always consider the electrode location and the experimental condition.

The rest of the paper is organized as follows: Section 2 presents a description of the system and its components, while Section 3 describes the system’s operation, including testing. In Section 4, various issues related to the proposed system (cost, power consumption, implementation issues, possible use for educational purposes, possible further improvements, etc.) are discussed. Finally, Section 5 concludes the article.

## 2. Description of the System’s Modules

### 2.1. Overview of the Proposed System

**Figure 1:**
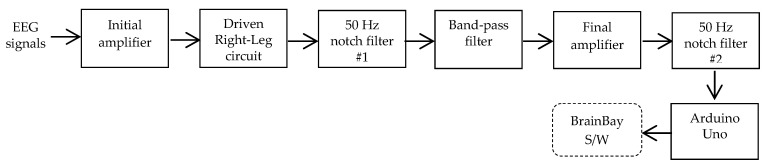
Block diagram of the proposed system.

The block diagram of the proposed system is shown in Figure 1. The EEG signals are extracted by means of proper electrodes and are, at first, processed by the system’s analog modules that amplify the EEG signals and, at the same time, reduce noise and interference. These modules are the initial amplifier, the driven right leg circuit (DRL), the first 50 Hz notch filter, the band-pass filter (that creates a 7–32.9 Hz pass-band capable of handling the alpha and beta signals), the final amplifier, and the final 50 Hz notch filter. The latter provides the final analog signal that is digitized by the Arduino Uno board. Finally, the derived digital signal is sent to a computer where it is analyzed by the open-source BrainBay (v2.8) software. Communication between the Arduino board and the computer can be implemented through either a wired or a wireless (WiFi) connection.

### 2.2. Electrodes and Input Interface

**Figure 2:**
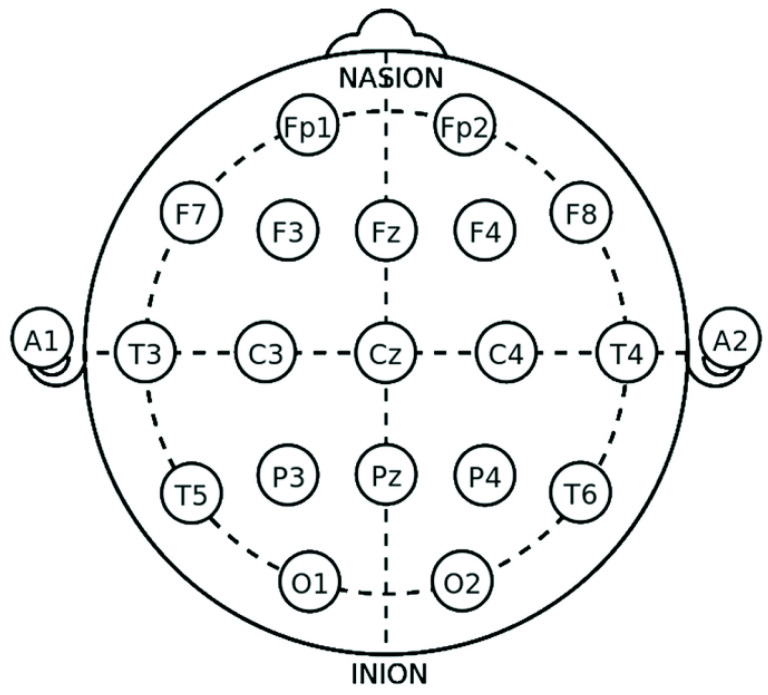
The placement of the electrodes according to the 10–20 scheme.

Two active and one reference passive electrode are used. The active electrodes are fixed at specific points of the head, following the 10–20 placement scheme (Figure 2). For the alpha signals of the occipital lobe, the electrodes are fixed at the Fp2 and O2 positions, while for the beta signals of the frontal lobe, the electrodes are fixed at the Fp2 and Fp8 positions. The electrodes are powered by a regulated voltage of +5 V provided by a low-dropout (LDO) regulator. The active electrodes are connected to the AD620 instrumentation amplifier (described in Section 2.3), while the passive electrode is connected to the driven right-leg (DRL) circuit described in Section 2.4.

### 2.3. Initial Amplifier

**Figure 3:**
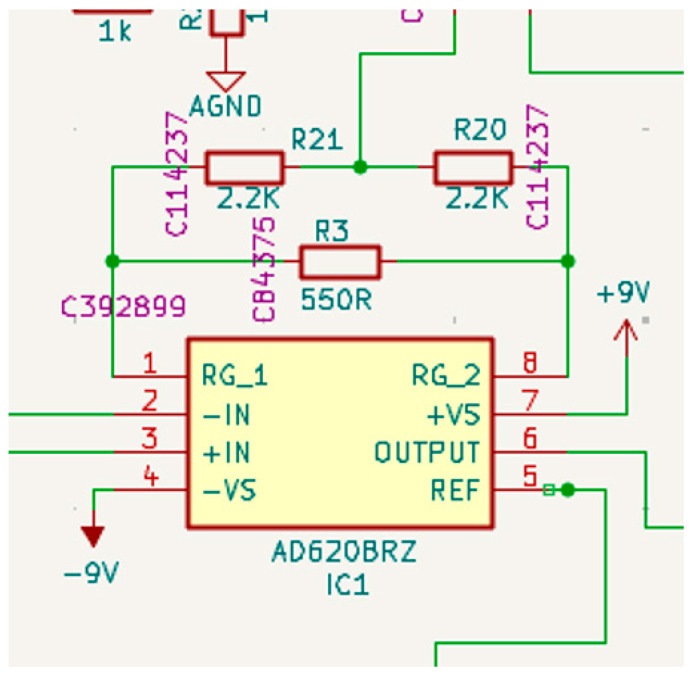
The initial amplification stage.

The electrode signals are first amplified by means of an AD620BRZ instrumentation amplifier ([18], Figure 3). This amplifier has been selected due to its high precision, low noise, and high stability, features that are considered necessary given the small value of the recorded EEG signals. Since the EEG signals are applied to the differential inputs of the amplifier, possible interferences (caused by, e.g., the power grid) are rejected. The amplification is equal to:

(1) $$G_{1}\;=\;1\;+\;\frac{49.4\;k\Omega }{RG}\approx 102,$$

where 49.4 kΩ represents the amplifier’s resistance while RG = R3 || (R1 + R2) = 488.9 Ω.

### 2.4. Driven Right-Leg (DRL) Circuit

**Figure 4:**
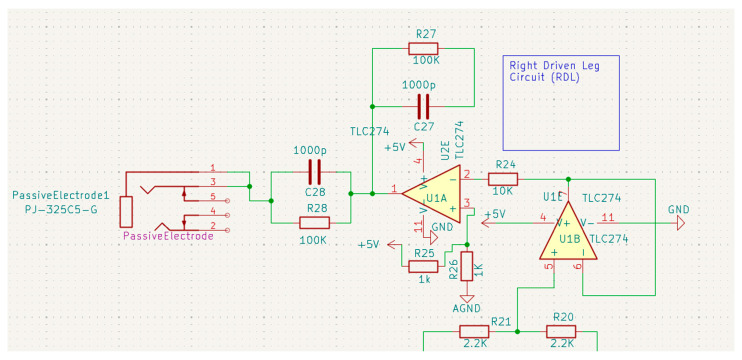
The DRL.

The DRL circuit (Figure 4) is crucial for bioelectrical signal acquisition systems since it minimizes common-mode interference and increases the electrical reference of the system. It has to be noted that the human body, apart from acting as an antenna that receives environmentally generated electromagnetic interference, also receives a significant amount of interference from the power grid (50 Hz). In this context, the DRL circuit detects the common-mode voltage (present at the inputs of the instrumentation amplifier) and sends it to the user’s body through the passive reference electrode. Thus, the DRL module “forces” the body to follow the reference potential created by the amplifier, effectively reducing the input common-mode voltage and increasing the common-mode rejection ratio (CMRR) of the overall system.

### 2.5. Initial Notch Filter

**Figure 5:**
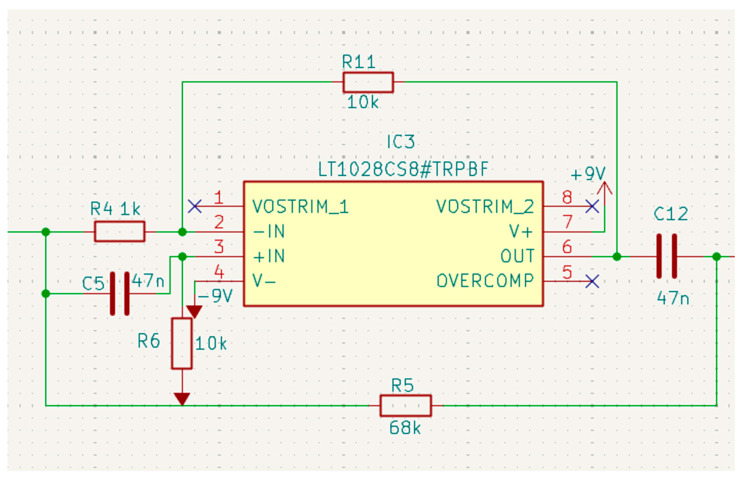
The initial 50 Hz notch filter.

**Figure 6:**
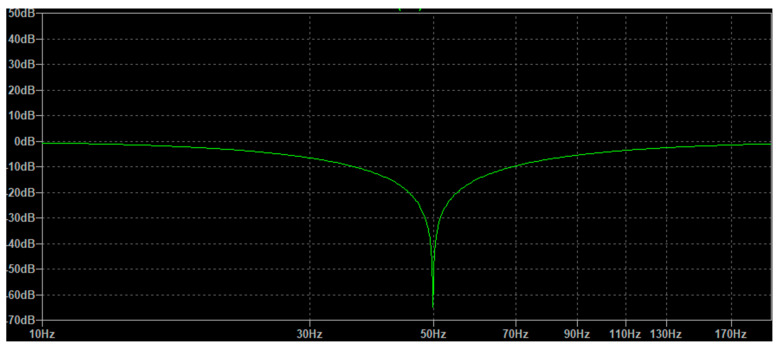
Magnitude of the transfer function of the 50 Hz notch filter.

The role of the 50 Hz notch filter (Figure 5) is to remove the noise created by the power grid, which is always present in biodynamic signals. Although the overall system operates with a fixed DC voltage source (9 V batteries), the power grid noise is still introduced through capacitive coupling with the environment and the human body. This is the reason why a notch filter is used at the early amplification stage. Apart from achieving a notch frequency equal to 50 Hz, the filter is designed in such a way as to achieve high stability and better behavior regarding signals of low amplitude (of the order of μV), such as the bioelectric ones. The magnitude of the transfer function of the filter is shown in Figure 6.

### 2.6. Band-Pass Filter

**Figure 7:**
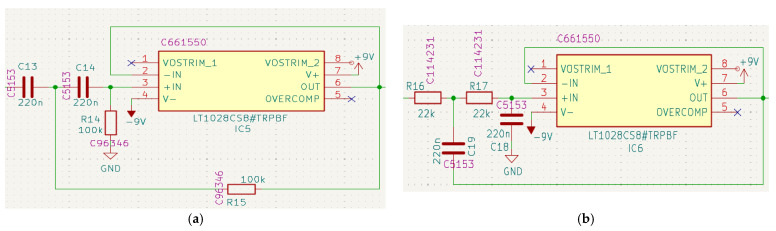
The band-pass filter: (**a**) the high-pass and (**b**) the low-pass components.

The band-pass filter (Figure 7) consists of two separate 2nd-order filters in series, a high-pass and a low-pass one, both based on the LT1208 amplifier [19]. The filter is capable of rejecting very-low-frequency components (such as DC offsets) as well as high-frequency components (electromagnetic noise, noise from muscular activity, etc.). The overall filter acts as a 4th-order one, providing a sharp attenuation outside its pass-band.

**Figure 8:**
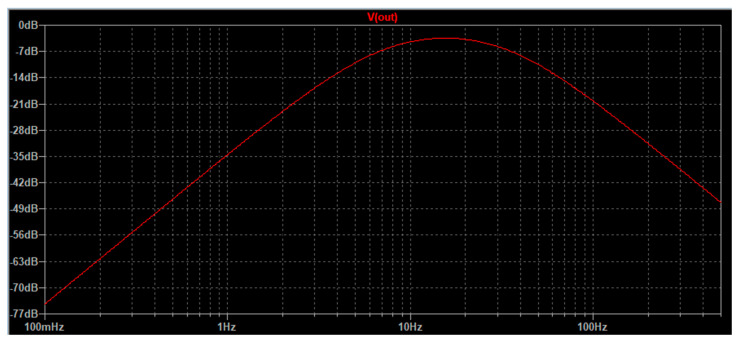
Magnitude of the transfer function of the band-pass filter.

The cut-off frequencies of the high-pass filter and the low-pass filter are given by Formulas (2) and (3) that, given the values of the respective resistors and capacitors of each filter, provide cut-off frequencies of 7.2 Hz (high-pass filter) and 32.9 Hz (low-pass filter). The above-mentioned frequencies, in combination with the fact that the filters’ design is based on the low-noise, high-input impedance and low-output impedance LT1208 amplifier [19], create a pass-band (Figure 8) that supports both alpha and beta brain signals and, at the same time, cuts off any noise that may occur:

(2) $$f_{\mathrm{HP}}\;=\;\frac{1}{2\pi \sqrt{R_{14}R_{15}C_{13}C_{14}}},$$

(3) $$f_{\mathrm{LP}}=\frac{1}{2\pi \sqrt{R_{16}R_{17}C_{18}C_{19}}}.$$

### 2.7. Final Amplification

The final amplification aims at bringing the alpha and beta signals to levels that are suitable for the signals’ digitization by means of the Arduino module.

**Figure 9:**
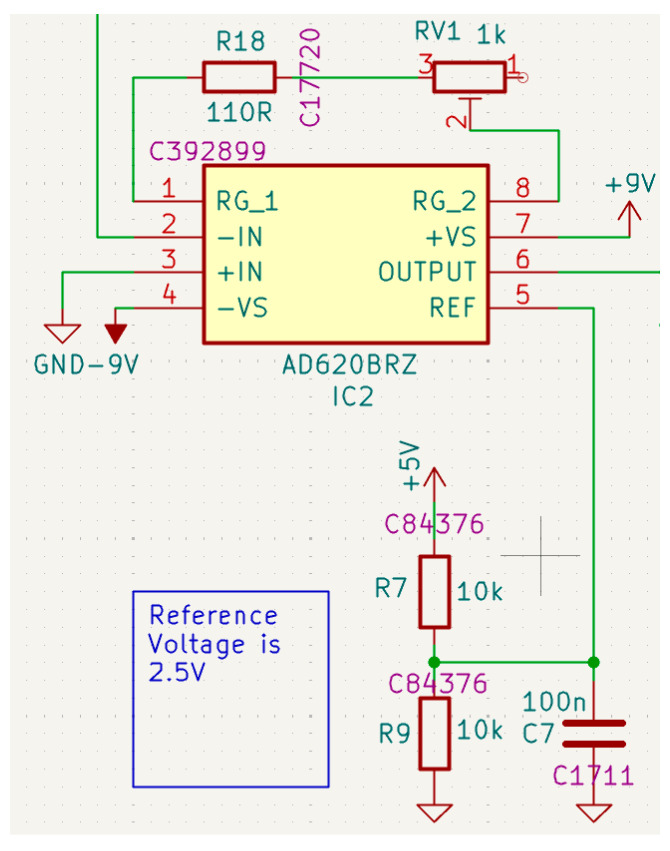
The final amplification stage.

In the sub-circuit shown in Figure 9, a reference voltage of 2.5 V is created at the REF pin of the AD620BRZ amplifier [18] by means of the voltage divider formed by the R7 and R9 resistors. This is the voltage around which the signal oscillates. The amplification of the amplifier is given by Formula (4), where RG consists of the R18 = 110 Ω resistor in series with a 1.1 kΩ potentiometer (0 ≤ R_pot_ ≤ 1.1 kΩ):

(4) $$G_{2}\;=\;1\;+\frac{49.4k\Omega }{\left(R_{G}+R_{pot}\right)}.$$

Given the above values, G_2_ is between 45.5 and 450.1, resulting in an overall amplification:

G_tot_ = G_1_G_2_, (5)

between about 4640 and 45,910. The final amplification also ensures that the phase of the amplified signal is that of the original one.

### 2.8. Final Notch Filter

**Figure 10:**
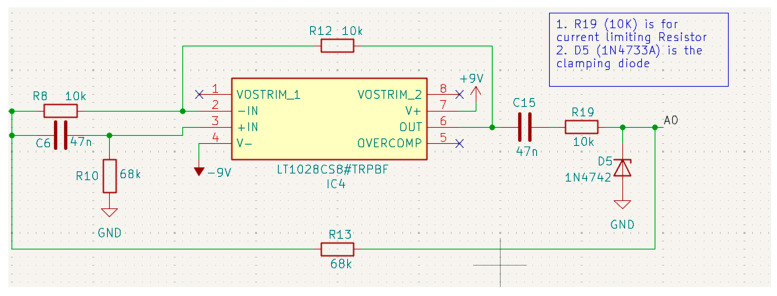
The final 50 Hz notch filter.

The role of the final 50 Hz notch filter (Figure 10) is to remove the noise caused by the power grid that may have entered due to possible electromagnetic interference and the high amplification described above. The filter (whose transfer function is similar to that of the initial notch filter) has a sufficiently deep notch to reject the 50 Hz frequency without affecting the useful EEG signal. At the filter’s output, a protection sub-circuit has been connected (including the resistor and a Zener diode) with the aim to protect the Arduino module from spikes and/or high-value unpredicted currents. In this way, the EEG signals are made ready for digitization with minimal network noise, appropriate amplitude, and increased electrical safety, providing the system with the required reliability for operation in actual experimental conditions.

### 2.9. Power Supply

In order to reduce interference from the power grid, the system is supplied power by two 9 V batteries, while a voltage regulator is used to provide a constant operating voltage of 5 V suitable for certain parts of the system. Decoupling capacitors are also used to filter high-frequency noise. Finally, a common grounding scheme is employed to minimize grounding loops.

### 2.10. The Arduino Unit

The role of the Arduino unit is the digitization of the analog signal delivered by the final notch filter (which is the amplified, noise-free version of the original alpha and beta signals).

**Figure 11:**
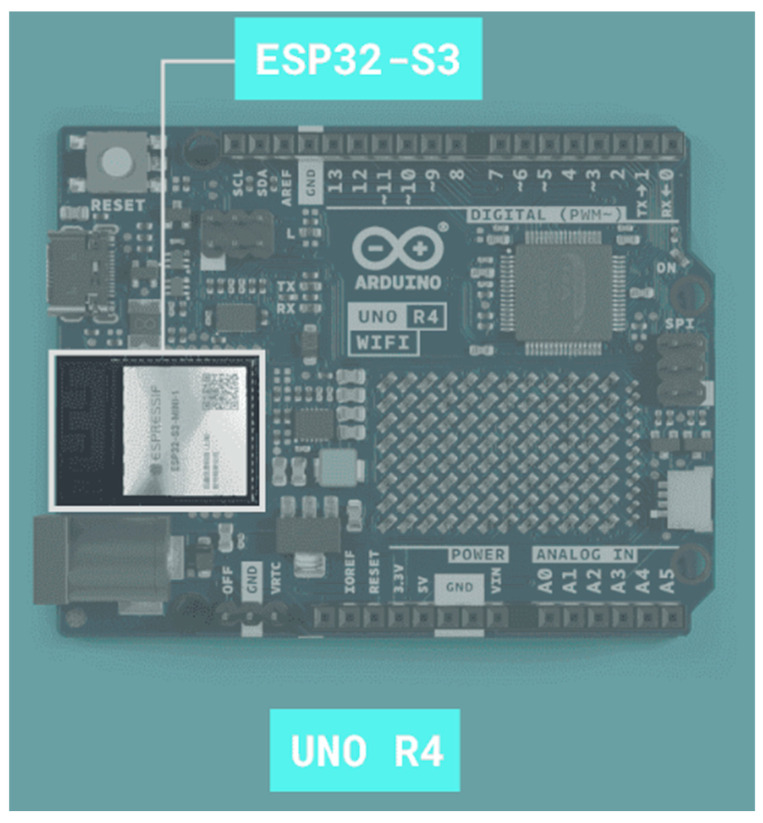
The Arduino Uno R4 WiFi unit [20].

The Arduino Uno R4 WiFi unit (Figure 11) uses the 32-bit RA4M1 microcontroller and the ESP32 module for WiFi and Bluetooth connectivity [20]. The unit has 6 analog input pins and 14 digital input/output pins, 6 of which can also be used as pulse-width-modulation (PWM) ports capable of simulating analog outputs. Each digital input/output involves digital signals with values of 0 or 5 V, while the analog signals have values between 0 and 5 V. The unit’s main technical characteristics are shown in Table 2. 

Programming of the module is performed by means of the “Wiring” language (a variation of C++), and the activation of the Arduino Uno functions is achieved through the IDE (v2.3.5). Apart from being openly available, the IDE is compatible with all the existing variants of Arduino boards and can run on Windows, Linux, and MacOS.

Another merit of Arduino is that the produced data can be processed by commonly used software tools/packages, such as Excel, MATLAB, and LabView. In the described system, the data have been processed by the open-source software BrainBay (v2.8).

## 3. System’s Operation

**Figure 12:**
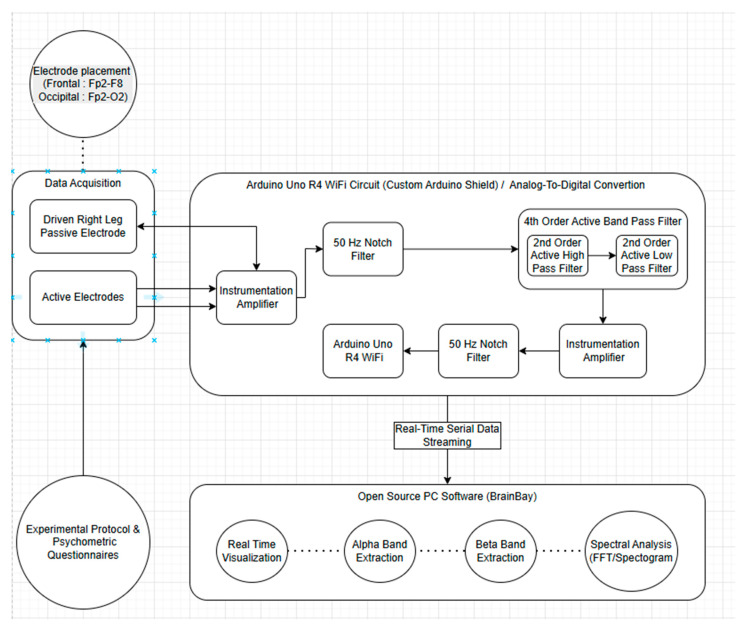
An operational diagram for the proposed system.

**Figure 13:**
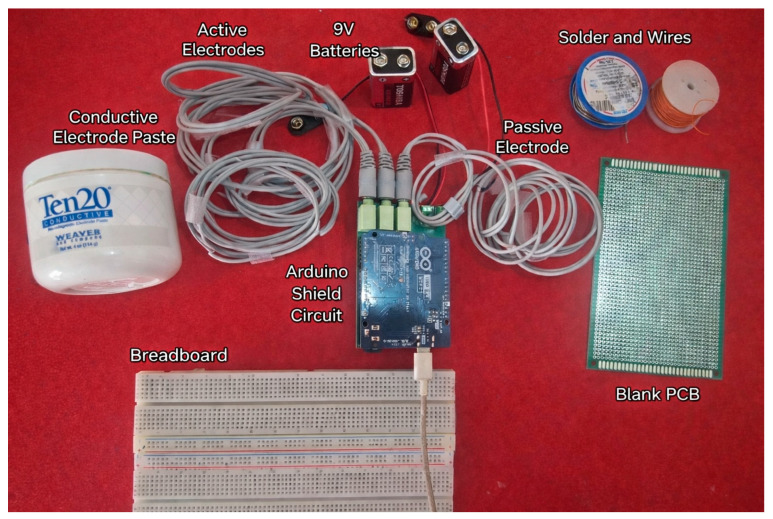
A photo of the actual prototype.

**Figure 14:**
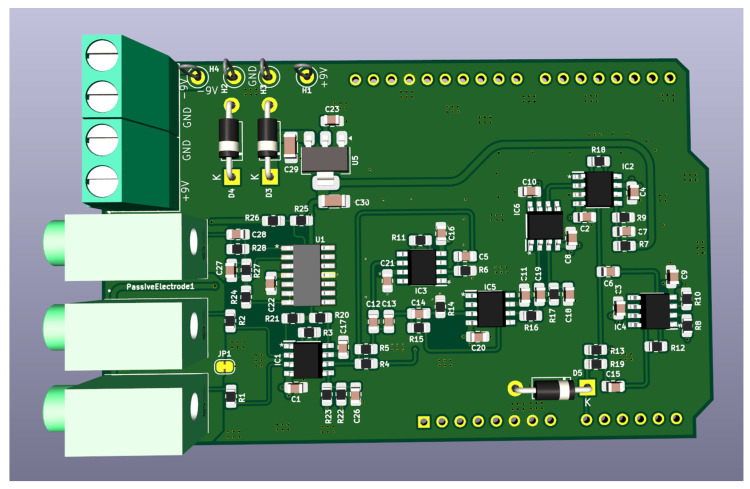
The PCB-based Arduino shield (simulation).

An operational diagram of the proposed system is illustrated in Figure 12, while a photo of the actual system is shown in Figure 13. The system’s implementation is based on a printed circuit board (PCB)-based Arduino shield (Figure 14) to take advantage of the PCB’s merits, including compactness and high density, environmental protection, and reduction of noise and electromagnetic interference.

As also explained above, the EEG signals are extracted by means of the electrodes and are first processed by the analog modules of the system, namely, the initial amplifier, the first 50 Hz notch filter, the band-pass filter (that creates a 7.2–32.9 Hz pass-band capable of handling the alpha and beta signals), the final amplifier, and the second 50 Hz notch filter. The final analog signal is digitized by the Arduino Uno board, which produces the digital signal to be processed by the open-source BrainBay software [21].

The testing of the proposed prototype mainly aimed at the proof-of-concept and, as a first step, it involved the recording and depiction (in both the time and the frequency domains) of the alpha signals in the following states:

(a) Resting state.
(b) Experimentally induced stress state (according to the Trier Mental Challenge Test—TMCT).
(c) Self-regulation/relaxation (with eyes closed) by means of a controlled breathing exercise (through controlled diaphragmatic breathing using the 4–7–8 breathing technique).

For reproducible EEG recordings, the participant should be seated comfortably in a quiet room with stable ambient lighting and minimal external auditory noise. During the recording, unnecessary movement, speaking, blinking, and facial muscle activity should be avoided as much as possible. The eyes-open or eyes-closed state should be explicitly reported because these two conditions can lead to different EEG power distributions, especially in the alpha band.

During the tests, the alpha signals of an adult person were depicted in both the time and the frequency domains. This set of measurements mainly aimed at verifying the proper operation of the proposed device and its ability to produce reliable results regarding the above-mentioned states. Electrical safety in those tests was in compliance with the requirements of the IEC-60601-1 standard [22,23].

At first, the alpha signal was recorded with the person being in resting state, with the aim for the recording to be used as a reference for the above-mentioned states (b) and (c). As an additional verification, the amplitude of the alpha signal was observed with the user having their eyes closed, and it was found to increase as expected.

**Figure 15:**
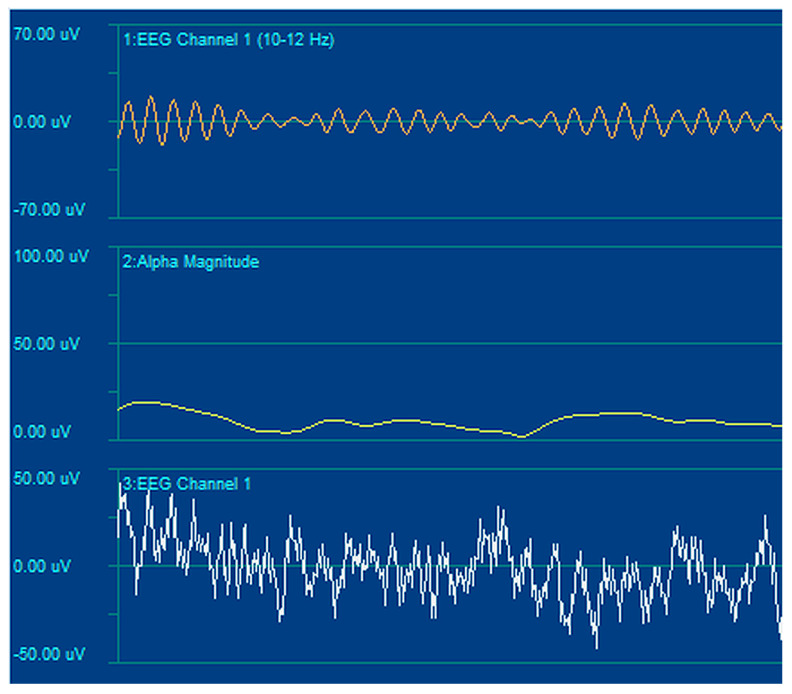
An oscillogram of the alpha signal with the individual being in resting state. The upper waveform represents the filtered alpha signal, the middle one illustrates the alpha signal’s amplitude, while the lower waveform depicts the raw version of the alpha signal. The time intervals where the signal’s amplitude appears increased correspond to the user having their eyes closed.

**Figure 16:**
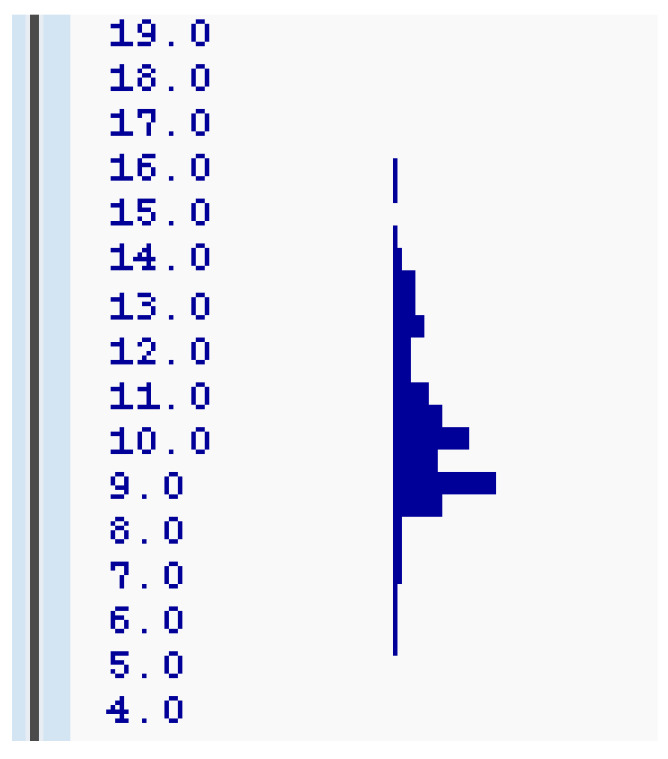
Spectral density of the alpha signal with the individual being in resting state.

A typical oscillogram regarding the alpha signal for the resting state is illustrated in Figure 15. The spectral density of the signal is depicted in Figure 16, which shows that the spectrum of the alpha signal covers the 7–13 Hz range, with most of the signal’s power appearing between about 9 and 11 Hz.

**Figure 17:**
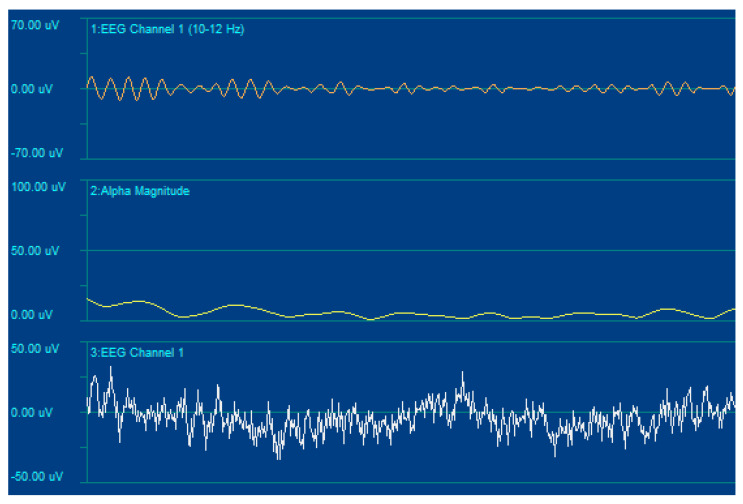
An oscillogram of the alpha signal with the individual being in a state of experimentally induced stress. As in Figure 15, the three waveforms represent the filtered alpha signal, the alpha signal’s amplitude, and the raw alpha signal. The alpha signal’s reduction (compared to the alpha signal of the resting state) is obvious in all three waveforms.

**Figure 18:**
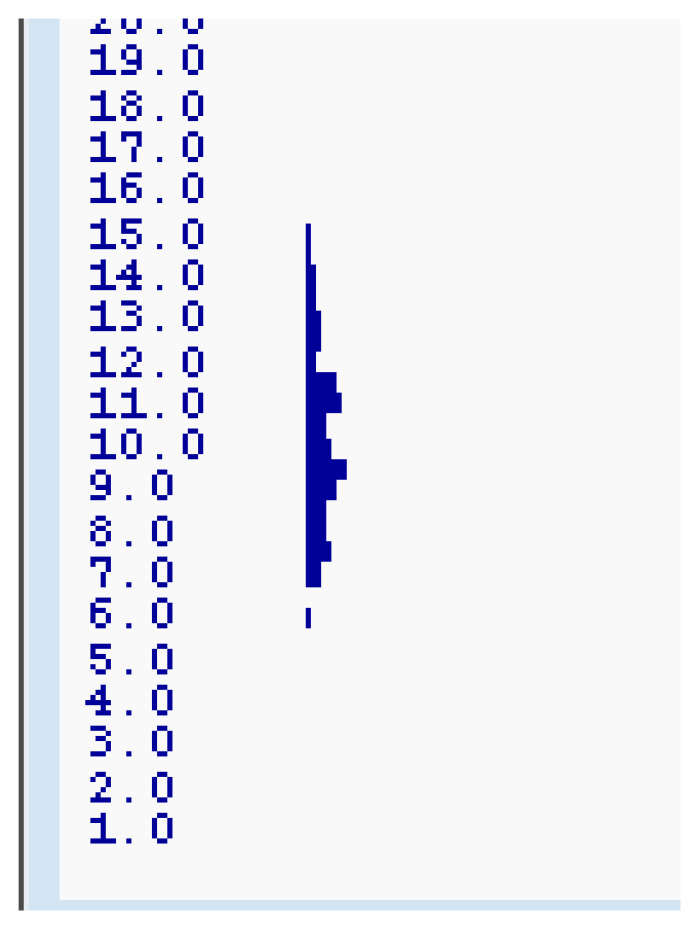
Spectral density of the alpha signal with the individual being in an experimentally induced stress state. Compared to the graph of Figure 16, the signal is weaker and almost uniformly spread over the 7–13 Hz frequency range.

Then, the alpha signal was recorded with the individual being in an experimentally induced stress state (Figure 17 and Figure 18). Apart from being weaker in amplitude, the signal power is almost uniformly spread over the 7–13 Hz frequency range.

**Figure 19:**
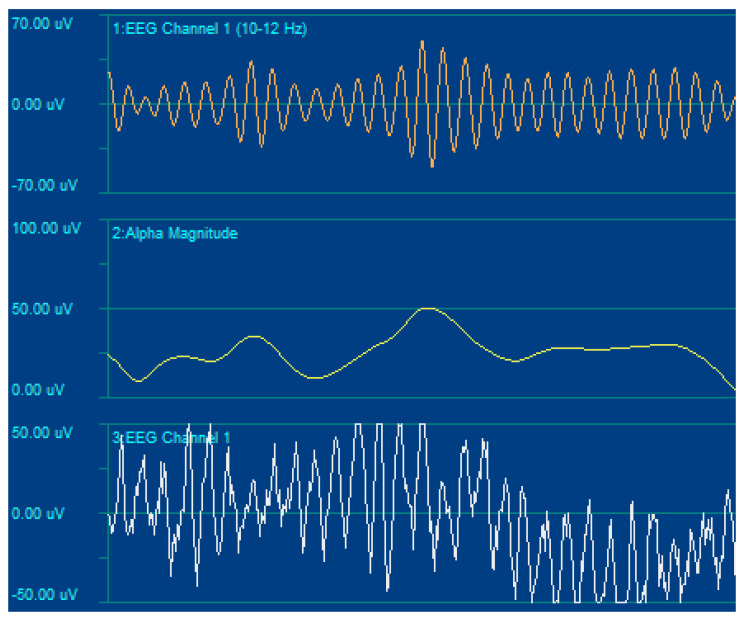
An oscillogram of the alpha signal after self-regulation/relaxation. As in Figure 15 and Figure 17, the three waveforms represent the filtered alpha signal, the alpha signal’s amplitude, and the raw alpha signal. The alpha signal’s increase compared to that of Figure 17 (as a result of the self-regulation/relaxation process) is evident.

**Figure 20:**
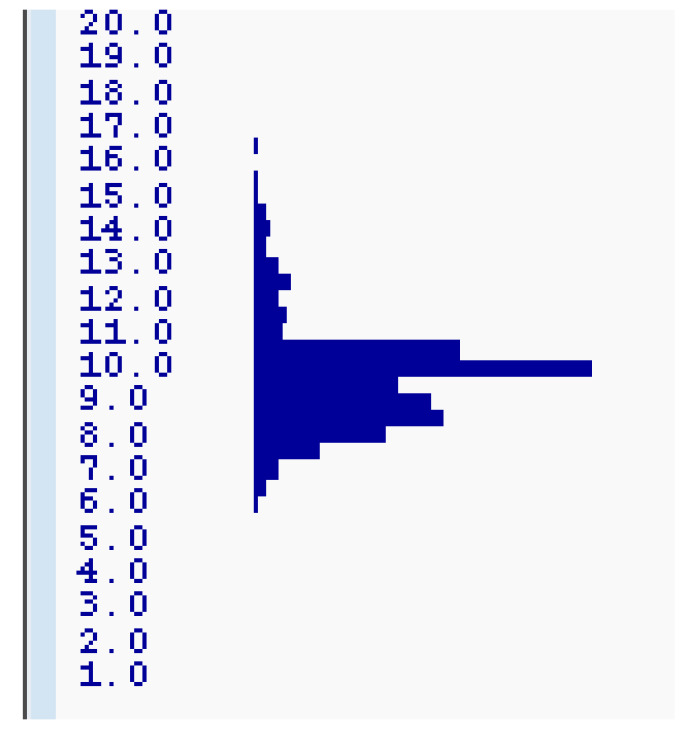
Spectral density of the alpha signal after self-regulation/relaxation.

Finally, the alpha signal was recorded immediately after a self-regulation process (involving control of breath and body awareness). The result was a strongly enhanced alpha signal, as it can be seen in the oscillogram of Figure 19 compared to the oscillograms of Figure 15 and Figure 17. A similar result regards the signal’s spectral density (Figure 20), which, compared to that for the resting and the induced stress states, appears considerably stronger and concentrated over the 8–11 Hz frequency range.

**Figure 21:**
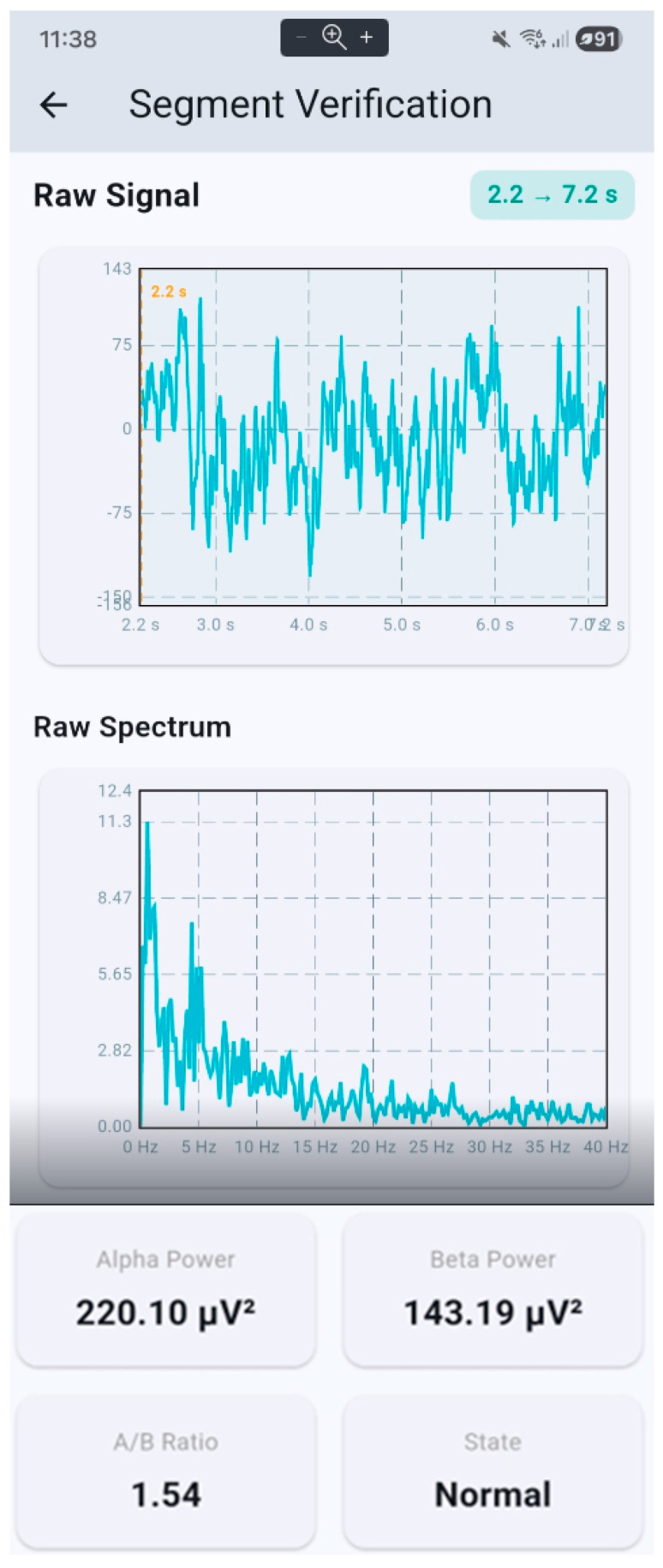
EEG segment and corresponding spectral density graph (together with relevant alpha- and beta-signal metrics) for the resting baseline condition.

**Figure 22:**
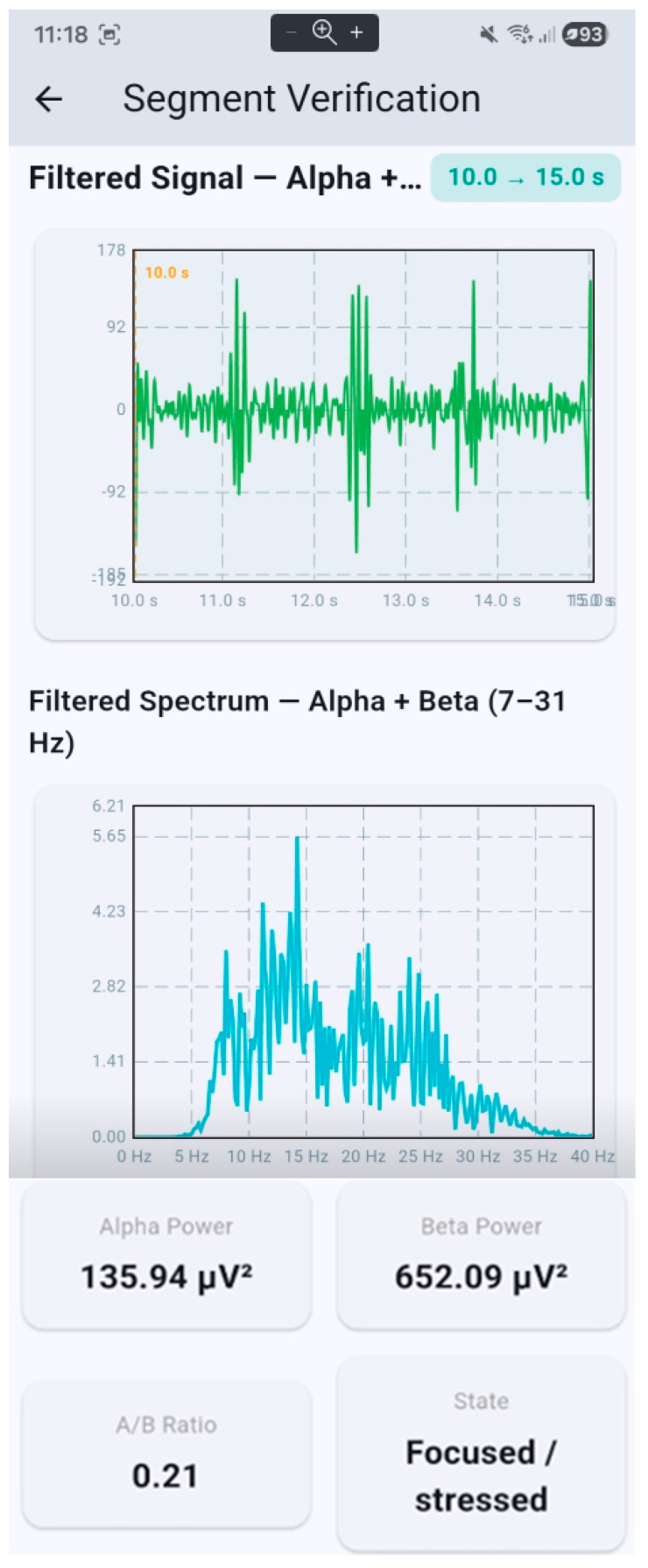
The oscillogram and the spectral density graph (7–31 Hz) of the filtered alpha and beta signals together with relevant alpha- and beta-signal metrics for the condition of experimentally induced stress. The decrease in the alpha-signal power, the increase in the beta-signal power, and the decrease in the alpha-to-beta ratio (in comparison with the respective values for the resting baseline condition) are evident.

**Figure 23:**
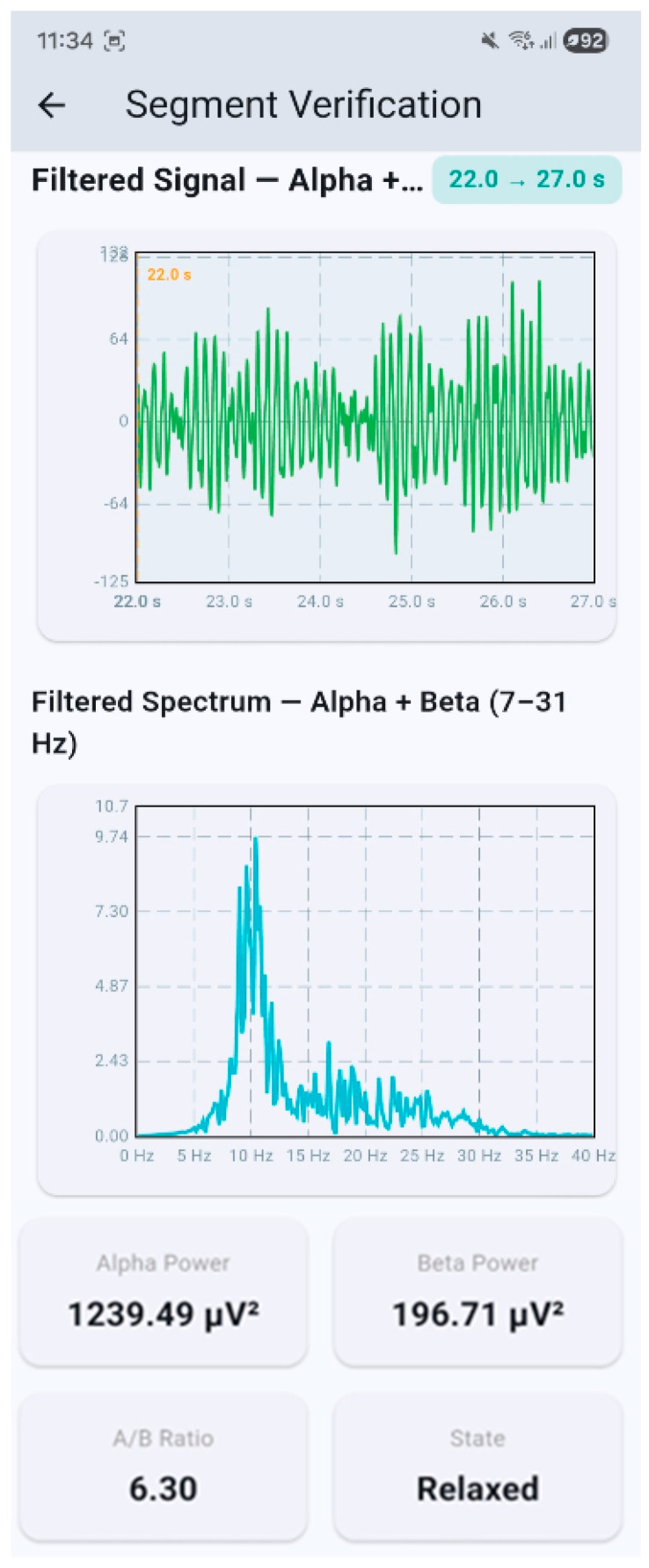
The oscillogram and the spectral density graph (7–31 Hz) of the filtered alpha and beta signals together with relevant alpha- and beta-signal metrics for the relaxation/eyes-closed condition. The increase in the alpha-signal power, the decrease in the beta-signal power, and the increase in the alpha-to-beta ratio are evident.

To validate the software of the proposed device and further verify the device’s proper operation, a mobile application for educational use was developed, which extends the hardware prototype by providing signal visualization, spectral analysis, segment verification, and alpha/beta metrics’ extraction. Publicly available EEG reference datasets [24,25,26] were used, and the results are shown in Figure 21, Figure 22 and Figure 23 and summarized in Table 3. Three states are presented: a resting baseline segment, an experimentally induced stress/task segment, and a relaxation/eyes-closed segment. The same analysis procedure was applied to all segments in order to extract alpha power, beta power, and the alpha-to-beta power ratio. The software structure also allows future expansion toward additional EEG-derived metrics, more advanced analysis methods, machine-learning-based processing, and the integration of an educational AI assistant.

## 4. Discussion

The system’s proper operation and ability to produce reliable results (with regard to normal and stressful conditions) were verified through the two sets of tests depicted in Figure 15, Figure 16, Figure 17, Figure 18, Figure 19 and Figure 20 and Figure 21, Figure 22 and Figure 23. It should be emphasized that EEG signals are influenced by multiple internal and external factors, such as age, fatigue, attention, emotional state, eye condition, lighting, acoustic noise, muscle activity, eye movements, and electrode placement. Therefore, the alpha-band and beta-band metrics should not be interpreted as unambiguous stress biomarkers and, in the present work, these parameters are only used as supportive indicators for self-monitoring, education, and exploratory analysis. In view of the above, the proposed prototype is not intended for clinical diagnosis or therapeutic decision-making but rather for capturing and presenting stress-related EEG indicators for self-use and/or educational purposes.

The cost of the proposed prototype is analyzed in Table 4. The overall cost is estimated to be about EUR 230, which is considered affordable and reasonable for this kind of system.

The estimated power consumption of the system is shown in Table 5.

Based on Table 4, the estimated total power consumption is about 1.2 to 1.8 W (with the Arduino’s WiFi off) or about 1.4 to 2.1 W (with the Arduino’s WiFi on). On the other hand, the total current of the system is about 130 and 203 mA (with the Arduino’s WiFi off) or about 170 to 258 mA (with the Arduino’s WiFi on). Given that the batteries used have a capacity of about 550 mAh, the operational time of the system is estimated to be about 550/203 = 2.7 h to 550/130 = 4.2 h (with the Arduino’s WiFi off) or about 550/253 = 2.1 h to 550/170 = 3.2 h (with the Arduino’s WiFi on), which is considered adequate even for a continuous operation.

The presented prototype can be considered to have a technology readiness level (TRL) equal to 4, which corresponds to the components and/or breadboard validation in a laboratory environment [27].

The presented prototype can also be used for didactical and learning purposes. Projects such as the one described above constitute rich educational activities in the sense that they require the combination of diverse knowledge domains and skills while involving cooperative and inquiry-based learning in accordance with the STEAM principles. This project in particular introduces students to terms and notions ranging from psychology and medicine to mathematics and technology (such as the brain’s electrical activity, basic signal concepts and processes, microcontrollers, and electronic components), while making them employ a diverse range of engineering and general skills (including integration of components into an operational prototype, evaluation of the final product, as well as time-planning and cost considerations). The project’s educational value can be highlighted either by demonstrating the system and helping the students comprehend its design, implementation, and operation or, as a more demanding assignment, by asking the students to design and/or rebuild parts or the whole of the system. In this context, the project comprises all three types of knowledge (content, pedagogical, and technological) of the TPACK framework [28] and all six levels (remember, understand, apply, analyze, evaluate, and create) of Bloom’s taxonomy [29]. This is particularly important for students who, after their graduation, would be employed as teachers in technology-oriented schools since it would make them capable of applying effective teaching/learning practices in their own classes.

Though portable, the proposed prototype is more suitable for indoor environments given that, in open spaces, noises could distort the EEG signals. The solution could be a system with improved components (e.g., dry electrodes and additional and higher-quality filters); that would, however, increase the cost.

Further actions on the presented prototype would include its complete validation regarding both the engineering and neurophysiological aspects. This should involve further measurements (e.g., the signal-to-noise ratio) as well as a quantitative comparison of power spectrum densities among resting, stress, and relaxation states. Regarding the neurophysiological validation (and given the rather nuanced relationship between the alpha and beta signals and stress situations), a more systematic testing, based on widely accepted protocols, should take place involving a larger sample and including the appropriate statistical analysis. Such a validation would provide further insight into the function of the proposed system and possibly expand its usability as a device that could help the user not only detect and self-monitor stress but also self-treat it.

Employment of machine learning techniques is also an option and can include, for example, supervised classification algorithms, such as support vector machine (SVM), random forests, or linear discriminant analysis (LDA) trained on extracted EEG features (band power, spectral ratios, and alpha peak frequency) to automatically distinguish between resting, stress, and relaxation states. Convolutional neural networks (CNNs) operating directly on raw or spectral EEG representations may further improve classification accuracy as larger datasets become available. Machine learning implementations should be accompanied by cross-validation, transparent reporting of performance metrics, and careful attention to inter-individual variability, in EEG-based stress recognition. The goal is a personalized, adaptive classifier capable of calibrating itself to the individual user’s baseline EEG profile, thereby improving both sensitivity and specificity of stress detection in real-world conditions.

A future development of the proposed prototype could anticipate a wearable version (with improved components to mitigate noise) that would also have the possibility of automatically transmitting a notification to the user once a reduced alpha/beta ratio (indication of a stress situation under development) is detected. That would allow the user to proceed to self-regulating/relaxing actions before the stress escalates.

## 5. Conclusions

Given that anxiety disorder and stress are factors that can severely affect health and quality of life, this article presented (in a proof-of-concept manner) a portable, low-cost Arduino-based prototype that, by recording the alpha and beta brain signals through EEG, enables the user to detect stress and mitigate it by means of self-regulating and relaxing actions. The system is fully battery-powered and has the possibility of wirelessly transmitting the EEG data. The proposed system is fully evaluated with regard to circuit characteristics, cost, power consumption, and operating time and is also treated as an educational tool associated with the STEAM and TPACK frameworks. During the tests, the described prototype was able to successfully demonstrate evident changes in the power of alpha and beta signals regarding the states of rest, induced stress, and biofeedback through self-regulation/relaxation. An improved version of the proposed system could also employ machine learning techniques, while, in the form of a wearable device, could possibly notify the user about a developing stress situation.

## Figures and Tables

**Table 1:** Human brain generated signals.

Signal	Typical Frequency Range (Hz)	Typical Amplitude (μV)	Comments
Delta	0.5–4	20–200	Deep sleep, low arousal, slow cortical activity
Theta	4–8	10	Drowsiness, memory processes, internal attention, cognitive control
Alpha	8–13	20–200	Relaxed wakefulness, closed-eyes resting state, reduced visual input, cortical idling/inhibition
Beta	13–30	5–10	Active attention, alertness, cognitive effort, sensorimotor processing, task engagement
Gamma	>30	5–10	Higher cognitive processing, perceptual binding, local cortical processing

**Table 2:** Technical characteristics of the Arduino Uno R4 WiFi unit [20].

Parameter	Value
Processor	RA4M1
Frequency	48 MHz
Analog inputs/outputs	6
Digital inputs/outputs	14
Memory size	256 KB flash + 1 KB EEPROM + 32 KB SRAM
Voltage	5 V
Dimensions	69 × 54 mm
Weight	23 g

**Table 3:** Metrics regarding the alpha and beta signals, as illustrated in Figure 21, Figure 22 and Figure 23.

Individual’s Condition	Alpha Signal Power (μV^2^)	Beta Signal Power (μV^2^)	Alpha/Beta Power Ratio
Resting baseline	220.10	143.19	1.54
Experimentally induced stress	135.94	652.09	0.21
Relaxation/eyes closed	1239.49	196.71	6.30

**Table 4:** Estimated cost of the proposed system.

Component	Estimated Cost (EUR)
Active electrodes (×2)	25.00
Passive electrode (×1)	5.00
AD620BRZ amplifiers (×2)	20.00
LT1028CS8 amplifiers (×4)	70.00
DRL	10.00
Arduino Uno R4 (×1)	40.00
Resistors (×30)	5.00
Capacitors (×20)	20.00
Jumpers, cables, etc.	10.00
PCB or breadboard	10.00
Gel (for the electrodes)	15.00
TOTAL COST	230.00

**Table 5:** Estimated power consumption of the proposed system.

Component	Voltage (V)	Current (mA) per Component	Quantity	Current (mA) ^1^	Power (mW) ^2^
Active electrodes	5	0.4–0.8	2	0.8–1.6	4.0–8.0
AD620BRZ amplifiers	18	1.3–1.5	2	2.6–3.0	46.8–54.0
LT1028CS8 amplifiers	18	8.5–11.0	4	34.0–44.0	612–792
DRL (TLC274)	5	2.8–6.4	2	5.6–12.8	28.0–64.0
Voltage divider (±9 V)	18	9.0	1	9.0	162
Voltage divider (5 V)	5	2.5	1	2.5	12.5
REF divider	5	0.3	1	0.3	1.5
Voltage regulator	5	5.0–10.0	1	5.0–10.0	25.0–50.0
Arduino Uno (WiFi off)	5	70–120	1	70–120	350–600
Arduino Uno (WiFi on)	5	110–175	1	110–175	550–875
TOTAL (WiFi off)	-	-	-	129.8–203.2	1241.8–1744
TOTAL (WiFi on)	-	-	-	169.8–258.2	1441.8–2019

^1^ Current = (current per component) × (quantity). ^2^ Power = (voltage) × (current per component) × (quantity).

## Data Availability

The original contributions presented in this study are included in the article. Further inquiries can be directed to the corresponding author.

## Abbreviations

The following abbreviations are used in this manuscript:

CMRR	Common-Mode Rejection Ratio
DRL	Driven Right Leg
EEG	Electroencephalogram
EEPROM	Electrically Erasable Programmable Read-Only Memory
IDE	Integrated Development Environment
LDO	Low Drop-Out
PCB	Printed Circuit Board
STEAM	Science, Technology, Education, Arts, and Mathematics
TMCT	Trier Mental Challenge Test
TRL	Technology Readiness Level
